# Lineage tracing shows that cell size asymmetries predict the dorsoventral axis in the sea star embryo

**DOI:** 10.1186/s12915-022-01359-3

**Published:** 2022-08-15

**Authors:** Vanessa Barone, Maria Byrne, Deirdre C. Lyons

**Affiliations:** 1 Center for Marine Biotechnology and Biomedicine, University of California San Diego, La Jolla, CA 92093 USA; 2 Bosch Institute and School of Life and Environmental Sciences, The University of Sydney, Camperdown, NSW 2006 Australia

**Keywords:** Cell fate, Cell size, Echinoderm, Dorsoventral axis, Embryo patterning

## Abstract

**Background:**

Cell size asymmetries are often linked to cell fate decisions, due to cell volumes and cell fate determinants being unequally partitioned during asymmetric cell divisions. A clear example is found in the sea urchin embryo, where a characteristic and obvious unequal 4th cleavage generates micromeres, which are necessary for mesendoderm cell fate specification. Unlike sea urchin development, sea star development is generally thought to have only equal cleavage. However, subtle cell size asymmetries can be observed in sea star embryos; whether those cell size asymmetries are consistently produced during sea star development and if they are involved in cell fate decisions remains unknown.

**Results:**

Using confocal live imaging of early embryos we quantified cell size asymmetries in 16-cell stage embryos of two sea star species, *Patiria miniata* and *Patiriella regularis*. Using photoconversion to perform lineage tracing, we find that the position of the smallest cells of *P. miniata* embryos is biased toward anterior ventral tissues. However, both blastomere dissociation and mechanical removal of one small cell do not prevent dorsoventral (DV) axis formation, suggesting that embryos compensate for the loss of those cells and that asymmetrical partitioning of maternal determinants is not strictly necessary for DV patterning. Finally, we show that manipulating cell size to introduce artificial cell size asymmetries is not sufficient to direct the positioning of the future DV axis in *P. miniata* embryos.

**Conclusions:**

Our results show that although cell size asymmetries are consistently produced during sea star early cleavage and are predictive of the DV axis, they are not necessary to instruct DV axis formation.

**Supplementary Information:**

The online version contains supplementary material available at 10.1186/s12915-022-01359-3.

## Background

Echinoid embryos, including sea urchin, heart urchin, pencil urchin and sand dollars, are unique among the echinoderms with respect to their visibly asymmetric cell division at the 4th cleavage: each vegetal blastomere of the 8-cell stage embryo will divide to produce one large cell—the macromere—and one very small cell—the micromere [1–3]. This asymmetric cell division has been best studied in the sea urchin embryo, where the unequal segregation of cell volumes is due to the spindle being positioned closer to the vegetal cortex, at the site of micromere formation [1, 4, 5]. The vegetal cortex, inherited by the micromeres only, is also enriched in cell fate determinants that induce differentiation into germ cells and primary mesenchyme cells [6–8]. The fact that both cell volumes and cell fate determinants are asymmetrically partitioned during the 4th cell division makes the micromeres the first morphological hallmark of the antero-posterior (AP) axis, with the micromeres positioned at the future posterior side of the embryo [2, 9, 10]. While cell fate determinants responsible for AP axis formation are also localized in the vegetal region of other echinoderm embryos, such as sea stars [11, 12] and brittle stars [13], cleavage is approximately equal in those species [14]. However, more subtle cell size asymmetries have been observed in the early embryos of asteroid sea stars [15], sea cucumbers [16] and feather stars [17], raising the questions of i) how consistent and stereotypical cell size asymmetries are and ii) if cell size asymmetries are relevant to the establishment of embryonic axes in echinoderm embryos other than the sea urchin. Here we answer these questions for the sea star embryo, using a combination of high-resolution live imaging, lineage tracing and micromanipulations to quantify cell size asymmetries and their role in axis formation.

Sea star embryos present holoblastic cleavage, with the first and second cleavages both aligned with the animal-vegetal axis and the third cleavage perpendicular to the first two, and dividing animal and vegetal halves of the embryo [15, 18, 19]. The animal-vegetal axis is established during oogenesis: the germinal vesicle is asymmetrically positioned in the immature oocyte, predicting both the site of polar body extrusion and the anterior side of the embryo [15]. Therefore, the AP axis of the sea star embryo can be identified already in the oocytes and its establishment is thought to depend on the asymmetric localization of maternal determinants [12, 15, 20, 21]. The first morphological hallmark of DV axis formation, instead, can be detected only at 3 days post fertilization (dpf), when the archenteron joins the anterior ectoderm to form the mouth on the ventral side of the embryo [15, 20].

Unlike sea urchins, early sea star embryos do not have obvious asymmetric cleavages and are generally thought to have equally sized cells [20]. However, to the best of our knowledge, measurements of cell volumes in the early sea star embryo have not been performed. We find more subtle, yet consistent, cell size asymmetries in 16-cell stage sea star embryos of two species, *Patiria miniata* and *Patiriella regularis*. Using lineage tracing, we show that the position of the smallest cells in *P. miniata* embryos is biased towards the anterior-ventral side of the future embryo. Using mechanical manipulations we determined that neither maternal determinants segregated in the smallest cells, nor cell size asymmetries per se, are required to induce embryonic axes, suggesting that although cell size asymmetries are consistently produced during sea star early cleavage they are not necessary to instruct DV axis formation.

## Results

In euechinoid sea urchins, the fourth cleavage is unequal and gives rise to 4 micromeres, 4 macromeres and 8 mesomeres [1–3]. In sea stars, cleavage is thought to be equal and all blastomeres at the 16-cell stage are expected to have similar size [20]. However, cell size asymmetries can be observed in a proportion of sea star embryos (Additional file 1: Fig S1, [15]).

**Fig. 1:**
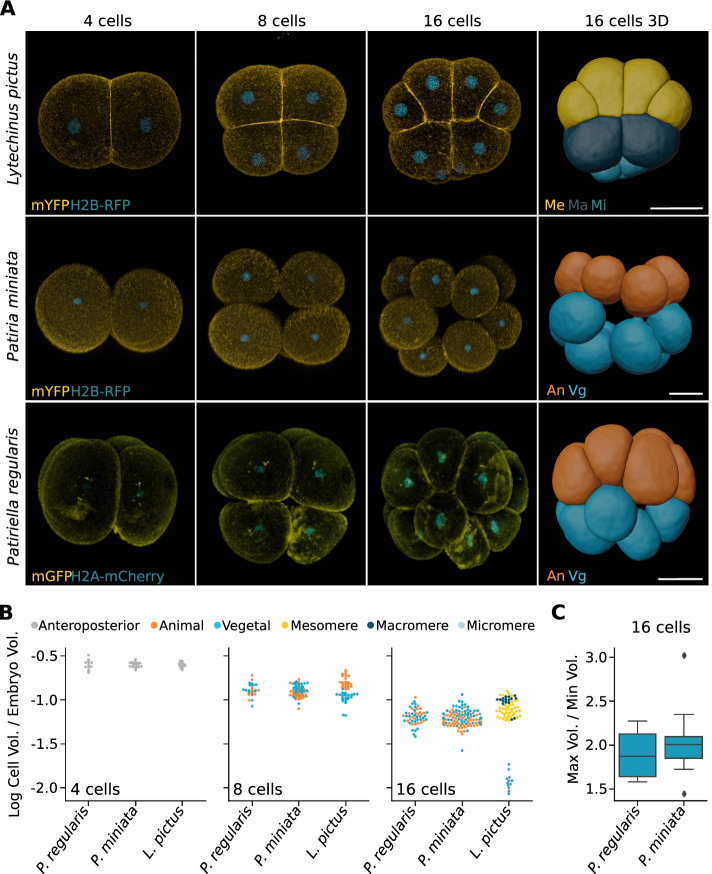
Cell size asymmetries in early sea star embryos. **A** Representative images of sea urchin (*Lytechinus pictus*) and sea star (*Patiria miniata*, *Patiriella regularis*) embryos at the 4-, 8- and 16-cell stages. Embryos were injected with mRNA coding for a membrane-bound fluorescent protein (mYFP, mGFP) and fluorescently tagged histone (H2B-RFP, H2B-mCherry) and subsequently imaged live on a confocal microscope. The datasets were segmented using the Fiji plugin Limeseg and individual blastomeres rendered as 3D meshes. Scale bars: 50 μm. **B** Volumes of individual blastomeres normalized to embryo volume, calculated as the sum of the volumes of the 4 blastomeres at the 4-cell stage. For sea star embryos, animal and vegetal poles were assigned according to the position of the polar bodies, and for sea urchin they were assigned according to the position of the micromeres. An, animal; Vg, vegetal; Me, mesomere; Ma, macromere; Mi, micromere. **C** Ratios of largest to smallest cells’ volume at 16-cell stage in sea star embryos. *L. pictus: n*=119 cells, 5 embryos. *P. miniata*: *n*=180 cells, 8 embryos; *P. regularis*: *n*= 91 cells, 4 embryos

This raises the possibility that cleavage of sea star embryos is not necessarily equal: it might produce less obvious, yet consistent, cell size asymmetries, possibly involved in axis determination. To test if sea star embryos present differently sized blastomeres and at what stage unequal cleavage might occur, we used high-resolution live imaging. We analyzed embryos of two sea star species (*Patiriella regularis* and *Patiria miniata*) and one euechinoid sea urchin species (*Lytechinus pictus*) for comparison. We performed high-resolution live imaging of embryos expressing membrane and nuclear markers from the 4-cell stage to the 16-cell stage (Fig. 1A; Additional file 2: Fig S2, Additional file 3: Movie S1, Additional file 4: Movie S2, Additional file 5: Movie S3). The animal pole was assigned as opposite to the site of formation of the micromeres in *L. pictus* and as the side of polar body extrusion in the sea stars. We subsequently segmented individual cells in 3D and measured cell volumes (Fig. 1A, B; Additional file 2: Fig S2). To compare cell size asymmetries across species with embryos of different sizes, cell volumes were normalized on embryo volume (Fig. 1B).

As expected, we found clear cell size asymmetries in 16-cell stage *L. pictus* embryos, with high volume variations between macromeres, mesomeres and micromeres (Fig. 1B). Interestingly, we also found variation within mesomeres, which showed a wide range of sizes (Fig. 1B).

The *P. regularis* embryos have higher variation in cell size compared to both *P. miniata* and *L. pictus* embryos at the 4-cell stage and variation similar to *P. miniata* at the 8- and 16-cell stages. Interestingly, the smallest cells tend to be vegetal in *P. regularis,* while they are mostly animal in *P. miniata* (Fig. 1B)*.* Notably, the differences between larger and smaller cells in sea star embryos at 16-cell stage are comparable to the differences between macromeres and mesomeres in the sea urchin (Fig. 1B), with the largest cells in *P. miniata* being on average twice the size of the smallest (max/min volume ratio: 2.05 ± 0.46; Fig. 1C). Taken together, these results suggest that cell size asymmetries are consistently produced during early cleavage of the sea star embryo. In *P. miniata*, even though there is no clear segregation between a group of large cells and a group of small cells, the majority of cells falling on the lower end of the spectrum of possible cell sizes are animal cells. This is similar to observations in *P. pectinifera* [15]. In *P. regularis,* instead, the smaller cells are mostly vegetal. This is different from the situation in echinoid species, in which the smallest cells, the micromeres, are always found on the vegetal side [2, 9, 10, 22, 23], where they are both necessary and sufficient to induce the AP axis [24]. The position of small cells between these two sea star species is, instead, variable with respect to the AP axis.

**Fig. 2:**
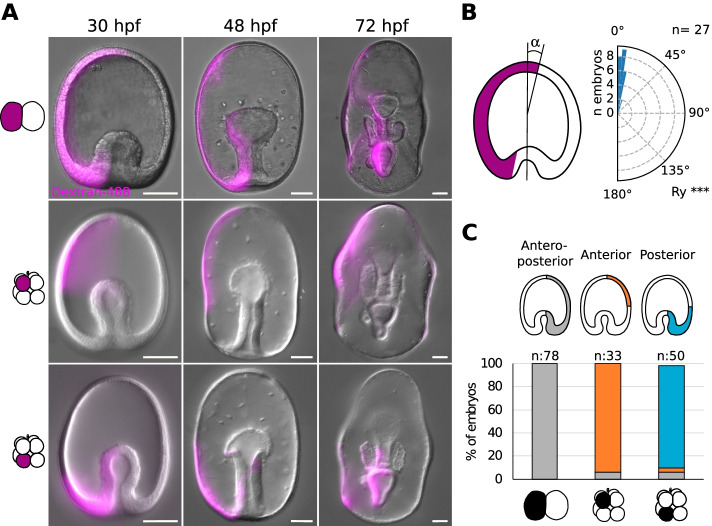
First and third cleavages predict the anteroposterior axis in *P. miniata* sea star embryo. **A** Representative images of *P. miniata* embryos injected with a lineage tracer at the 2- or 8-cell stage. One blastomere was injected with a mixture of Dextran-Alexa488 and mRNA coding for Histone-BFP either at the 2- or at the 8-cell stage. At the 8-cell stage, either one animal or one vegetal blastomere was injected, scored according to the position of the polar bodies. Embryos were then raised at 16C and imaged on an epifluorescence microscope at 30, 48 and 72 hpf. Scale bars: 50 μm. **B** Alignment of the first cleavage with the animal-vegetal axis. Embryos injected at the 2-cell stage were stained with cell mask orange and imaged in toto on a confocal microscope. The images were rendered in 3D and the angle formed between the clone and the animal-vegetal axis was measured. *n*= 27 embryos. Rayleigh test, ***: *p*-value < 0.001. **C** Quantification of the lineage-tracing experiments. One blastomere of *P. miniata* embryos was injected either at the 2- or 8-cell stages, discriminating between animal and vegetal blastomeres at the 8-cell stage based on the position of the polar bodies. Embryos were raised at 16C and the position of the injected clone scored at 26 hpf. *n*= 161 embryos, 4 experiments

**Fig. 3:**
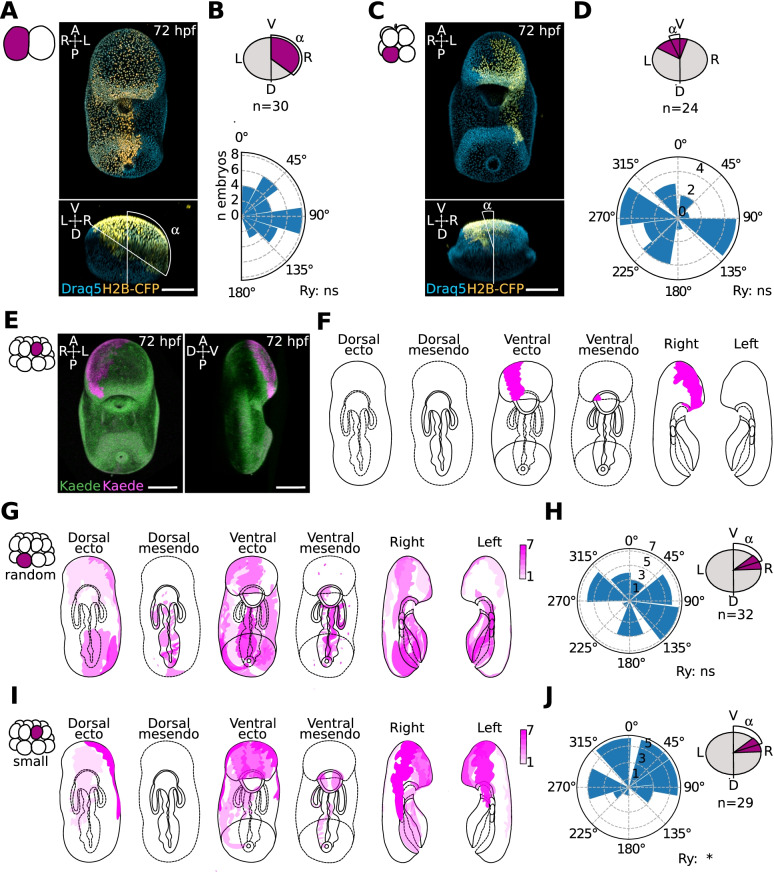
The position of smaller cells at 16-cell stage is biased toward the ventral side in *P. miniata* sea star embryos. **A**–**D** First and third cleavage in relation to the dorsoventral axis. Representative confocal image of a sea star larvae injected at the 2-cell stage (**A**) or 8-cell stage (**C**). One blastomere was injected with a mixture of Dextran-Alexa488 and mRNA coding for Histone-BFP at the 2-cell stage, raised at 16C, fixed at 72 hpf, stained with Draq5 to visualize all nuclei and imaged in toto on a confocal microscope. Quantification of the angles formed by the injected clone and the sagittal plane of the larva at 72 hpf is shown in **B** (2-cell; *n*= 30 embryos) and **D** (8-cell; *n*= 24 embryos). **E**–**L** Position of small cells in relation to the dorsoventral axis. (**E**) Representative confocal image of a sea star larva showing the clone derived by a small cell at the 16-cell stage. Oocytes were injected with mRNA coding for the photoconvertible protein Kaede (  ) and incubated ON at 16C. Oocytes were subsequently activated, fertilized and incubated until the 16-cell stage, when one of the 16-cell was photoconverted (  ) on a confocal microscope (405 nm laser). Embryos were raised at 16C for 72 hpf and then imaged live in toto on a confocal microscope. **F** Schematic representation of the clone shown in (**E**). Several views of the same embryo are drawn, to fully represent the position of the labelled clone in 3D. **G**–**J** Quantification of the positions of clones observed after the photoconversion of a random cell (**G**) or a small cell (**I**) at 16-cell stage and the angles of the same clones formed with the sagittal plane at 72 hpf (**H**, **J**). Random cells: *n*= 32 embryos; Small cells: *n*=29 embryos. Rayleigh test, *: *p*-value < 0.05; ns, not significant. Scale bars: 100 μm

Next, we asked if the position of small cells is predictive of DV axis formation in the sea star embryo. To test this hypothesis we first analyzed the relationship between cleavage planes and embryonic axes in *P. miniata*. In sea stars, the embryo develops into a blastula and gastrulation consists of invagination of mesendodermal cells at the vegetal side (opposite to the polar bodies) [15]. During gastrula stages, the archenteron elongates within the hollow ectoderm tissues and eventually joins the anterior ectoderm to open the mouth [15, 19]. The opening of the mouth, which for *P. miniata* happens at around 72 h post fertilization (hpf), is the first clear morphological hallmark of DV axis formation: the larva is now a bipinnaria and both mouth and anus open on the ventral side [15, 25, 26]. To confirm that first and third cleavages predict the AP axis of *P. miniata* larvae, we performed lineage tracing at the 2- and 8-cell stages (Fig. 2). We injected one cell with a fluorescent dextran and raised the injected embryos up to the bipinnaria stage. As expected, we found that in embryos injected at the 2-cell stage, the labelled clone constituted roughly half of the larvae, including anterior ectoderm, posterior ectoderm and mesendoderm tissues (Fig. 2A). We then measured the angle formed by the labelled clone with the animal-vegetal axis at the beginning of gastrulation (26 hpf) and found that the first cleavage aligns consistently with the animal-vegetal axis (Fig. 2B). In embryos injected in one animal blastomere at the 8-cell stage, the labelled clone included only a portion of anterior ectoderm, while in those injected into one vegetal blastomere the labelled clone included a portion of posterior ectoderm and mesendoderm (Fig. 2A, C). Next, we sought to analyze the position of the labelled clones with respect to the DV axis (Fig. 3A–D). To this aim we imaged 72 hpf larvae in toto on a confocal microscope, rendered the acquired images in 3D and virtually oriented the larva to achieve an anterior view, with the ventral side facing up (Fig. 3A, C). This allowed us to faithfully measure the angle between the DV axis and the labelled clones (Fig. 3B, D). We found that both first (Fig. 3A, B) and second cleavage (Fig. 3C, D) are positioned randomly with respect to the DV axis.

Taken together, these results show that the first cleavage is aligned with the animal-vegetal axis but is not predictive of the future larval DV axis. The third cleavage separates the embryo into animal and vegetal halves, with animal cells giving rise to anterior ectoderm and vegetal cells giving rise to posterior ectoderm and mesendoderm. This confirms what was shown for *P. pectinifera* [15] and our preliminary observations in *P. regularis* (Additional file 6: Fig S3).

To test if the cell size asymmetries arising during cleavage stages are predictive of the DV axis in *P. miniata,* we turned to an imaging approach. The appearance of small cells is most obvious at the 16-cell stage in this embryo: given the difficulty of faithfully injecting those smaller cells we used photoconversion to label them. We raised embryos expressing the photoconvertible protein Kaede to the 16-cell stage and then used a UV laser on a confocal microscope to photoconvert either a random cell (control) or one small cell (Additional file 7: Fig S4). We aimed at photoconverting the smallest cells of an embryo, independently of its animal or vegetal position: we selected the smallest cell once on the confocal microscope, by (1) imaging the whole embryo, (2) identifying two or three small cells and (3) measuring the three major axes of those cells to make sure the smallest was photoconverted. We then raised the photoconverted embryos to the bipinnaria stage and assessed the position of the photoconverted clones (Fig. 3E, F; Additional file 8: Fig S5, Additional file 9: Fig S6, Additional file 10: Movie S4). We found that in larvae where a random cell had been photoconverted, the labelled clones were positioned randomly with respect to both the AP and DV axes (Fig. 3G, H; Additional file 8: Fig S5). In larvae where one small cell had been photoconverted, the position of labelled clones was biased toward the ventral half of the anterior ectoderm (Fig. 3I, L; Additional file 9: Fig S6).

**Fig. 4:**
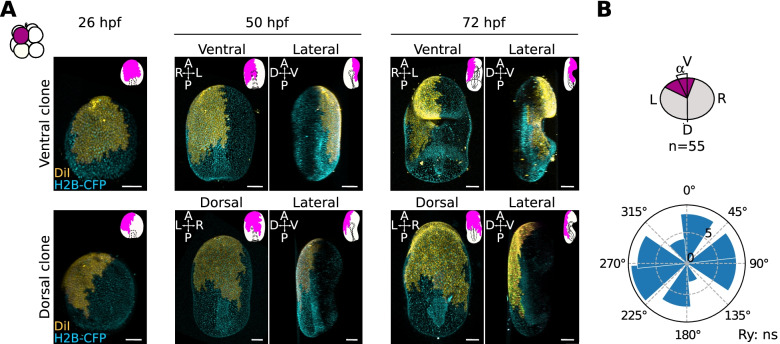
The position of animal clones is not biased with respect to DV axis due to morphogenesis. Representative confocal image of a sea star larvae injected at the 8-cell stage (**A**). Oocytes were injected with H2B-CFP to mark nuclei, fertilized and raised until the 8-cell stage, when one animal blastomere was injected with DiI. Embryos were imaged in toto on a confocal microscope at three different developmental stages (26, 50 and 72 hpf). Orientation of the embryo is indicated in the upper left corner of each image and a schematic representation of the clone is provided in the upper right corner. **B** Quantification of the angles formed by the injected clone and the sagittal plane of the larva at 72 hpf. *n*= 55 embryos. Rayleigh test, ns, not significant. Scale bars: 50 μm

It is possible that the observed ventral bias in the positioning of small cells may be due to a general bias of animal pole cells to give rise to ventral tissues, due to morphogenetic events. To test this possibility, we marked one random animal blastomere at the 8-cell stage and scored the position of the same labelled clone at three different stages: 26 hpf, early gastrula, when the apicobasal axis is most obvious; 50 hpf, late gastrula, when the bending of the anus allows identifying the ventral side of the gastrula but the mouth has not yet formed; and 72 hpf, bipinnaria, when the mouth, pre-oral hood and ciliary bands have formed (Fig. 4A; Additional file 11: Fig S7). We found that the clones derived from randomly labelled animal pole cells at the 8-cell stage are randomly positioned with respect to the dorsoventral axis (Fig. 4B; Additional file 11: Fig S7). Moreover, labelling of ventral clones shows that the ectodermal tissue forming the pre-oral hood is located on the future ventral side before mouth formation, and analysis of the position of several clones shows that the apical most tissues in the early gastrula correspond to the anterior-most tissues at the bipinnaria stage (Fig. 4A; Additional file 11: Fig S7). This experiment suggests that there is no bias in the positioning of animal pole clones due to morphogenesis.

Taken together, these results show that the smallest cells of *P. miniata* embryos at the 16-cell stage are more likely to arise in the animal side of the embryo, where the future ventral side will be specified. However, the association between the position of the smallest cell and the DV axis is broad, with clones deriving from the smallest cells found across the entire ventral half on the 3 dpf larvae, instead of exclusively at the site of mouth opening.

Next, we sought to understand if and how smaller cells may influence DV axis formation, as cell size asymmetries are often linked to cell differentiation due to asymmetric partitioning of both maternal determinants and cytoplasmic volumes during cell division (reviewed in [27]).

**Fig. 5:**
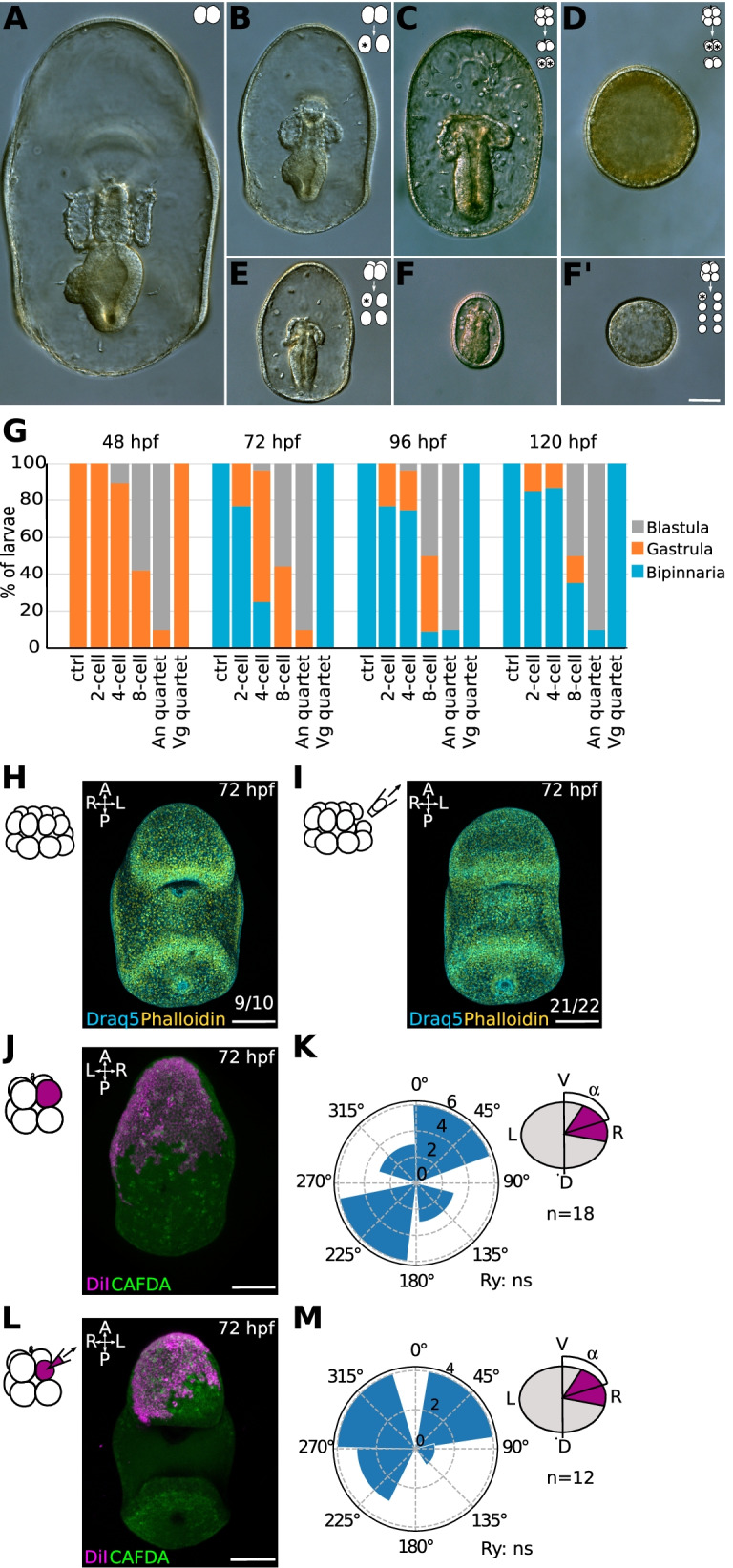
Cell size asymmetries are not necessary to establish the dorsoventral axis in *P. miniata* embryos. Representative DIC images of 72 hpf larvae generated by blastomeres of dissociated embryos. **A** CTRL embryos (not manipulated). **B**, **E**, and **F** Larvae generated by Individual blastomeres of embryos dissociated at the 2- (**B**), 4- (**E**) or 8- (**F**, **F’**) cell stages. **C**, **D** Larvae generated by vegetal (**C**) or animal (**D**) quartets of blastomeres isolated at the 8-cell stage. This corresponds to the vegetal and animal halves of an embryo. Vegetal or animal identity was established according to the position of the polar bodies. **G** Phenotypes of larvae formed by isolated blastomeres at between 48 and 120 hpf. Blastula: no invagination; Gastrula: evident gut; Bipinnaria: mouth formed. *n*= 94 isolated blastomeres; 35 embryos; 2 experiments. **H**, **I** Removal of one small cell at the 16-cell stage. Zygotes were denuded of their fertilization envelope and raised at 16C until the 16-cell stage. No cell (**H**) or one small cell (**I**) was removed by micropipette aspiration. Embryos were raised at 16C to 72 hpf, fixed, stained with Draq5 and Phalloidin and imaged on a confocal microscope. Phenotype quantification shown on bottom right. *n*=33 embryos, 2 experiments. **J**–**M** Cell size reduction. Representative confocal images of CTRL (**J**) or manipulated (**L**) larvae. One blastomere at the 8-cell stage was injected with DiI and immediately reduced in size by micropipette aspiration of cytoplasm. CTRL embryos were injected but not aspirated. Quantification of the angles formed by the injected clone and the sagittal plane of the larva at 72 hpf is shown in **K** (CTRL; *n*=18 embryos) and **M** (reduced; *n*= 11 embryos). Rayleigh test, ns, not significant. Scale bars: 50 μm

To test if asymmetric partitioning of maternal determinants is necessary to DV axis determination in *P. miniata,* we performed early embryo dissociations. We isolated blastomeres at the 2, 4 and 8-cell stages and raised them to the bipinnaria stage (Fig. 5; Additional file 12: Fig S8). As expected, control, whole embryos reached the bipinnaria stage at 72 hpf (Fig. 5A, G). Blastomeres isolated at the 2-cell stage formed half-sized larvae and reached the bipinnaria stage at 72 hpf in 79.6% of cases and by 120 hpf in 84.6% of cases (Fig. 5B, E, and G). Blastomeres isolated at the 4-cell stage formed smaller larvae and reached the bipinnaria stage at 72 hpf in 25% of cases and by 120 hpf in 86.9% of cases (Fig. 5B, E, and G). This suggests that in most cases all 4 blastomeres have the potential to form the DV axis.

To test if maternal determinants for DV axis formation are inherited by the animal cells at the 8-cell stage, we split embryos at the 8-cell stage into animal and vegetal quartets; we found that all the vegetal quartets developed into half-sized bipinnaria by 72 hpf (Fig. 5C, G), while animal quartets failed to form mesendoderm tissues and remained blastulae for 5 days (Fig. 5D, G). This suggests that the vegetal blastomeres retain the potency to establish a DV axis, even in the absence of animal cells. To test if all vegetal blastomeres are capable of establishing a DV axis, we isolated individual blastomeres at the 8-cell stage (Fig. 5F, F’). We found that 44.4% of the isolated blastomeres formed small gastrulae at 72 hpf (Fig. 5F) and 55.5% formed small blastulae (Fig. 5F’), indicating that isolated vegetal blastomeres can form mesendoderm tissues but fail to open a mouth by 72 hpf. However, 70.5% of those gastrulating mini-larvae reached the bipinnaria stage by 120 hpf, when the experiment was terminated (Fig. 5G; Additional file 12: Fig S8).

Taken together these results suggest that the vegetal portion of the embryo is necessary and sufficient for gut formation and establishment of the DV axis, although vegetal blastomeres isolated at the 8-cell stage establish a DV axis with considerable delay. Therefore, *P. miniata* and *P. regularis* (see Additional file 6: Fig S3) are similar to most other echinoderms analyzed so far in that differential allocation of maternal determinants is involved in the determination of the AP axis, but not necessary for the determination of the DV axis.

Zygotic cell fate determinants necessary for DV axis formation might accumulate in the small cells of *P. miniata* embryos and it is possible that cell fate determinant asymmetries are re-established after blastomeres dissociations. To test if zygotic determinants enriched in the small cells are necessary for DV axis formation, we used micropipette aspiration to remove one small cell from 16-cell stage *P. miniata* embryos (Fig. 5H–I). We found that perturbed embryos opened a mouth at 72 hpf, similar to CTRL non-perturbed embryos (Fig. 5H, I). Taken together these results suggest that enrichment of cell fate determinants in the small cells plays a negligible role in the determination of the DV axis in *P. miniata*.

Next, we sought to test if the relationship between smaller cells and DV axis formation in *P. miniata* may be due to cell size alone. If that were true, even after the smallest cells of an embryo were removed, either by aspiration or by dissociation, cell size asymmetries would still be in place, and the next smallest cell might instruct the position of the DV axis. To test this possibility, we artificially created a population of small cells on one side of the embryo, by removing cytoplasm from one of the animal blastomeres at the 8-cell stage by micropipette aspiration until that blastomere was the smallest in the embryo (Fig. 5J–M). We then scored the position of the clone formed by the progeny of that miniaturized blastomere at 72 hpf. We found that miniaturized clones were randomly positioned with respect to the DV axis (Fig. 5J, K), similar to CTRL embryos, in which one animal blastomere at the 8-cell stage was injected but not manipulated (Fig. 5L, M). Taken together these results indicate that manipulating cell size to introduce artificial cell size asymmetries is not sufficient to direct the positioning of the future DV axis in *P. miniata* embryos.

## Discussion

### Lineage tracing and fate mapping in *P. miniata*

To understand the role of cell size asymmetries in sea star embryonic development, we performed lineage tracing at 2-, 8- and 16-cell stages in *P. miniata*, as well as embryo dissociations at 2-, 4- and 8-cell stages. These experiments show that the relationships between the orientations of cleavage planes and the embryonic axes in *P. miniata* are similar to what was shown for *P. pectinifera* [15]. In both *P. miniata* and *P. pectinifera*, the first cleavage is aligned with the animal-vegetal axis but is not predictive of the future larval DV axis and the third cleavage separates the embryo into animal and vegetal halves, with animal cells giving rise to anterior ectoderm and vegetal cells giving rise to posterior ectoderm and mesendoderm (Figs. 2, 3 A–D, and Fig. 4) [15]. Moreover, our dissociation experiments show that the potential of forming bipinnaria larvae, i.e. of establishing both AP and DV axes, is retained by both blastomeres at the 2-cell stage and all four blastomeres at the 4-cell stage, but only by the four vegetal blastomeres at the 8-cell stage (Fig. 4A–G; Additional file 11: Fig S7). Therefore, the vegetal portion of the embryo is necessary and sufficient for gut formation and establishment of the DV axis in *P. miniata*, although vegetal blastomeres isolated at the 8-cell stage establish a DV axis with considerable delay. It is worth noting that these results contradict Dan-Sohkawa and Satoh [18], who showed all blastomeres isolated at the 8-cell stage form gastrulae in *P. pectinifera*, but in agreement with Maruyama and Shinoda [11] who showed only the vegetal blastomeres form gastrulae in that same species. Interestingly, Maruyama and Shinoda find that all isolated blastomeres at the 2- and 4-cell stages, and all of the vegetal blastomeres isolated at the 8-cell stage, form bipinnaria, although they do not discuss the timing of these events. Our results also align with recent experiments showing that the vegetal-most portion of cytoplasm in the oocyte is necessary for gut induction in *P. miniata* [12]. Therefore, *P. miniata* is similar to most other echinoderms analyzed so far in that differential allocation of maternal determinants is involved in the determination of the AP axis, but not strictly necessary for the determination of the DV axis.

**Fig. 6:**
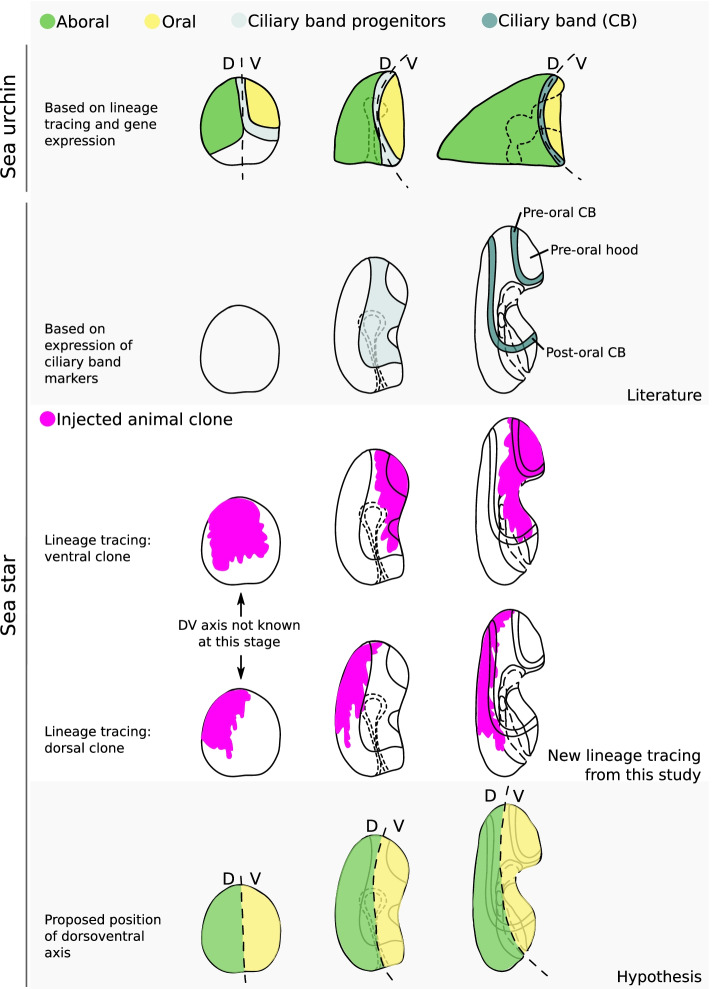
Differences in ectoderm morphogenesis between sea urchin and sea star. Schematic representation of the known and hypothesized fate maps of sea urchin and sea star ectoderm. In the sea urchin, the ciliary band marks the boundary between dorsal/aboral and ventral/oral ectoderm (scheme adapted from Davidson et al., 1993). In the sea star, the position of the precursors of the ciliary band (Yankura et al., 2013) and the location of pre-oral and post-oral ciliary bands are known (Nakajima et al. 2004; Hinman and Burke 2018). Our lineage tracing data suggests that the boundary between dorsal/aboral and ventral/oral anterior ectoderm lies between the two ciliary bands in the sea star

Interestingly, lineage tracing of animal clones at the 8- and 16-cell stages shows that morphogenesis of the anterior ectoderm differs between sea star and sea urchin. In the blastula of planktotrophic sea urchin species, the progenitors of the ciliary band are positioned at the boundary between dorsal and ventral ectoderm tissues (Fig. 6) [2, 9, 28–34]. The morphogenetic events that transform the gastrula into the sea urchin pluteus larva cause the dorsal ectoderm to expand anteriorly and ventrally, while the ventral ectoderm, where the mouth forms, folds inward (Fig. 6) [14, 29]; all the while, however, the ciliary band remains at the boundary between dorsal (also called aboral) and ventral (also called oral) ectoderm (Fig. 6) [14, 29]. Therefore, if a dorsal/aboral animal blastomere of an 8-cell stage sea urchin embryo is marked, the clone derived from that labelled cell includes roughly half of the ciliary band and the ectoderm dorsal to the ciliary band [9]. If a ventral/oral animal blastomere is marked, the derived clone will include roughly half of the ciliary band and the ectoderm ventral to the ciliary band [9]. The lineage tracing experiments presented here suggest that the situation is different in *P. miniata* embryos: the boundary between dorsal/aboral and ventral/oral clones does not lie along the ciliary bands.

In the bipinnaria larva of *P. miniata* and other planktotrophic sea star species there are two ciliary bands, one that loops around the anterior ectoderm only and is positioned dorsally to the mouth (pre-oral ciliary band, Fig. 6; [35–37]) and one that loops around both anterior and posterior ectoderm, passing ventrally between the mouth and the anus (post-oral ciliary band, Fig. 6; [35–37]). We find that the anterior-most boundary of labelled animal clones lies between the two ciliary bands in *P. miniata* (Figs. 2, 3, and 4; Additional file 8: Fig S5, Additional file 9: Fig S6, Additional file 11: Fig S7). Following the same labelled clones over time, and scoring their position before and after the formation of mouth and ciliary bands, we find that the position of those clones with respect to the dorsoventral axis does not change dramatically (Figs. 4 and 6; Additional file 11: Fig S7). These results support the notion that the ectoderm tissue encircled by the pre-oral ciliary band originates from ventral/oral ectoderm [35, 36] and pave the way for further experiments aimed at clarifying the relationships between the dorsoventral axis and ciliary band formation in sea star embryos.

### Cell size asymmetries in the sea star embryo

Species-specific cleavage patterns producing cell size asymmetries that are related to cell fate determination are common among metazoans [27, 38, 39]. Among echinoderms, cell size asymmetries have been best-studied in echinoids; however, cell size asymmetries of varying degrees have been reported in embryos of most classes of echinoderms, including asteroids, holothuroids, and crinoids [15–17]. In the sea urchin embryo, a specific protein, Activator of G-Protein Signaling, drives the asymmetric cell division that generates micromeres and macromeres, due to an evolutionarily novel motif that recruits mitotic spindles to the vegetal cortex [5]. That motif is absent in other echinoderm species and it is sufficient to induce asymmetric cleavage in the sea star embryo [5]. The generation of micromeres in echinoids is therefore a highly controlled event that has evolved due to modifications in the sequence of key regulatory proteins.

In contrast, it is generally thought that cell size asymmetries in echinoderms other than echinoids arise randomly, possibly due to the accumulation of small asymmetries across several cleavages, and are not linked to cell fate decisions, nor to axes determination. These hypotheses, however, had never been tested. Here we provide evidence that cell size asymmetries arise consistently during early development of asteroid sea star embryos. The position of smaller cells is non-random, is species-specific and is biased toward anterior ventral tissues in *P. miniata.* However, removal of the smaller cells does not affect development: sea star embryos can compensate for the loss of those cells, showing that asymmetric deposition of cell fate determinants in those cells is not strictly necessary for DV patterning. Moreover, the position of the DV axis is not strongly biased by experimental reduction of cell size, indicating that cell size asymmetries alone are not sufficient to instruct the positioning of embryonic axes. This situation may represent an evolutionary “in-between”, with cell size asymmetries arising consistently, but not necessarily being linked to cell fate decisions. It will be interesting to expand the analysis of cell asymmetries to other echinoderm species, with the potential of identifying other evolutionary states, ranging from completely randomly produced cell size asymmetries to other examples, more similar to the sea urchin, where differences in cell sizes are linked to cell fate decisions.

## Conclusions

This study provides extensive lineage tracing of the sea star ectoderm, showing that (i) the position of small cells at cleavage stages predicts the future ventral side of the sea star larva and (ii) the boundary between dorsal and ventral ectoderm lies between the two ciliary bands in the sea star. The results presented open the way for future work on the evolution of cell size asymmetries and their relationship with embryonic axis specification.

## Methods

### Animal husbandry

Adult *Lytechinus pictus* were collected at La Jolla, CA, and held in free-flowing seawater aquaria at a temperature of 16C. Spawning was induced by injection of 0.5M KCl, as previously described [40]. Adult *Patiria miniata* were purchased from Monterey Abalone Company (Monterey, CA) or South Coast Bio-Marine LLC (San Pedro, CA) and held in free-flowing seawater aquaria at a temperature of 12-16C. Adult *Patiriella regularis* were collected off the coast of Tasmania (Australia) and held in aquaria at a temperature of 20C. Sea star gametes were obtained as previously described [41]. Briefly, ovaries and spermogonia were dissected via a small incision on the ventral side of adults. Sperm was stored undiluted at 4C while ovaries were fragmented to release oocytes in FSW. Maturation of released oocytes was induced by incubating for 1h at 16C in 3 μM 1-Methyladenine (Fisher Scientific, 5142-22-3). All embryos were raised in 0.22 μm filtered seawater (FSW) with the addition of 0.6 μg/ml Penicillin G sodium salt (Millipore Sigma, P3032) and 2 μg/ml streptomycin sulphate salt (Millipore Sigma, S1277).

### mRNA, dextran and Dil Injections

mRNAs were synthesized with the mMessage mMachine SP6 Transcription Kit (Invitrogen, AM1340). To label entire embryos, *Lytechinus pictus* were injected at the 1-cell stage with a mix of mRNAs coding for membrane-bound YFP and Histone-2B-RFP (mYFP, 50 ng/μl; H2BRFP 400 ng/μl). *Patiria miniata* and *Patiriella regularis* immature oocytes were injected with mRNAs. mRNAs were injected, alone or in combination, to label membranes (mYFP or mGFP, 400 ng/μl), nuclei (H2B-RFP, 400 ng/μl; H2A-mCherry, 400 ng/μl) or cytoplasm (Kaede, 400 ng/μl). Injected oocytes were incubated at 16C overnight, activated and fertilized. To label individual blastomeres at the 2-, 4- or 8-cell stages, one blastomere of *Patiria miniata* and *Patiriella regularis* embryos was injected with either mRNAs coding for H2B-CFP (100 ng/μl), H2A-mCherry (400 ng/μl), Dextran Oregon Green 488 (1 mg/ml; Invitrogen, D7171) or DiI (Life Technologies, D282).

### Whole embryo staining

To label whole embryos for live imaging, embryos at 48 or 72 hpf were incubated with either CMFDA (1:2000, Invitrogen, C2925) or Cell Mask Green (1:10,000, Invitrogen, C37608) for 30 min at RT. To label actin and nuclei, embryos at 24, 48 or 72 hpf were fixed in 2% Paraformaldehyde (PFA) at 4C overnight (ON) and stained with Alexa Fluor™ 488 Phalloidin (Invitrogen, A12379), DRAQ5 (Invitrogen, 65-0880-92) or DAPI (Invitrogen, D1306).

### Early embryo cell volumes

*Lytechinus pictus* embryos were injected with mRNAs coding for membrane and nuclear markers on a glass bottom dish (MatTek, P35G-1.5-14-C) coated with protamine, incubated at 16C until the 2-cell stage and then imaged on an inverted Leica Sp8 confocal microscope (20× objective, NA 0.7, 16C controlled temperature) until the 16-cell stage. *Patiria miniata* and *Patiriella regularis* embryos expressing membrane and nuclear markers were mounted on a glass bottom dish. No medium was used to immobilize the embryos: the glass bottom part of the dish was covered with a coverslip and sealed with vaseline. This creates a 1 mm deep chamber in which capillarity prevents the embryos from moving, until they develop cilia. Additional FSW was added in the dish, to help with temperature control. Embryos can be cultured in such chambers without apparent defects up to 3 dpf, when they would start feeding. Embryos were incubated until the 2-cell stage and then imaged on an inverted Leica Sp8 confocal microscope (20X objective, NA 0.7, 16C controlled temperature for *Patiria miniata*) or Zeiss LSM 800 confocal microscope (20X Objective, NA 0.8, 20C controlled temperature for *Patiriella regularis*) until the 16-cell stage. Datasets were 3D rendered using Imaris 6.4 (Bitplane), and further analyzed using the Fiji plugin Limeseg [42] and custom python scripts. Briefly, 3D meshes for individual blastomeres at the 4-, 8- and 16-cell stages were computed with the Limeseg plugin, exported in .ply format and their volume was calculated in python. To compare cell sizes across species, cell volumes were normalized on embryo volumes, calculated as the sum of the volumes of the 4 cells at the 4-cell stage for each embryo analyzed.

### Lineage tracing

To establish how early cleavage planes relate to the AP axis, one blastomere of 2- or 8-cell stage *Patiria miniata* embryos was injected with Dextran-488. Embryos injected at the 8-cell stage were separated into two groups immediately after injections based on the position of the injected blastomere: close to the polar body was considered animal, and opposite to polar bodies was considered vegetal. Embryos were raised to 72 hpf and the position of the labelled clone was scored at 30, 48 and 72 hpf. A subset of embryos was live imaged at the same stages with an upright Zeiss Imager M2 (20X objective, NA 0.7). To establish how early cleavage planes are related to the DV axis, one blastomere of 2- or 8-cell stage *Patiria miniata* embryos were injected with mRNA coding for a nuclear marker. Embryos were raised until the 72 hpf, fixed, stained with DRAQ5 and imaged on an inverted Leica Sp8 confocal microscope (20× objective, NA 0.7). Datasets were 3D rendered with Imaris 6.4 (Bitplane) to measure the angle between the labelled clone and the sagittal plane. To determine how the position of the smaller cells in *Patiria miniata* relates to the DV axis, embryos expressing the photoconvertible protein Kaede were raised until the 16-cell stage, mounted on a glass bottom dish and imaged with an inverted Zeiss LSM 710 confocal microscope (20X objective, NA 0.8). Embryos are oriented randomly with this method and the cell that happened to be in optimal position for photoconversion, i.e. tilted so as not to photoconvert other cells above it, was photoncoverted using the bleaching tool in the Zeiss Blue software: 405 nm laser at 1% power emission was used to scan a ROI inside the target cells for up to 60 sec. As control (CTRL), any embryo was photoconverted; as experimental condition (small cell), only embryos in which the smallest cell was in the optimal position were photoconverted. The smallest cells were identified by measuring the three major axes compared with the other cells of the embryo. Photoconverted embryos were recovered, raised until 72 hpf and imaged live on an inverted Leica Sp8 confocal microscope (20× objective, NA 0.7). Datasets were 3D rendered using Imaris 6.4 (Bitplane) and the larvae were virtually oriented to achieve an anterior view with the ventral side up, allowing us to measure the angle between the photoconverted clone and the sagittal plane. Schematic representations of all clones were drawn based on the 3D renderings to identify clones that formed similar structures in multiple larvae. Several views were drawn for each embryo, as well as for ectodermal and mesendodermal tissues, to provide a full representation of the 3D position of the labelled clone.

### Mechanical manipulation of sea star embryos

To perform embryo dissociations, *Patiria miniata* oocytes were fertilized in 10 mM 4-Aminobenzoic acid (PABA, Sigma, A9878) in FSW and embryos were incubated in 10 mM PABA in FSW for 1 hpf. The fertilization envelope was mechanically removed by passing the embryos through a narrow glass pipette. Denuded embryos were transferred to gelatin-coated dishes and raised in FSW until the 2-, 4- or 8-cell stage. Blastomeres were separated using an entomology needle and/or passing the embryos through a narrow glass pipette coated with gelatine. Individual blastomeres were raised separately and their phenotypes scored at 48, 72, 96 and 120 hpf. A subset of dissociated embryos was live imaged at the same stages on an upright Zeiss Imager M2 microscope (20X objective, NA 0.8). To remove one small cell at the 16-cell stage, denuded embryos were cultured in FSW on gelatine dishes and manipulated with glass micropipettes connected to a syringe. Two micropipettes were used, one with an opening diameter of 80 μm to orient and hold the embryo and the second with an opening diameter of 20 μm to remove one small cell by suction. The manipulated embryos were raised until the 72 hpf, fixed, stained with Phalloidin-488 and DRAQ5 and imaged on an inverted Leica Sp8 confocal microscope (20X objective, NA 0.7). To create a population of artificially small cells, one animal blastomere at the 8-cell stage was injected with DiI and part of its cytoplasm was suctioned away using a glass micropipette with an opening of about 5 μm. CTRL (injected but not reduced) and reduced embryos were raised until the 72 hpf stage, stained with either Cell Mask Green or CMFDA and live imaged on an inverted Leica Sp8 confocal microscope (20× objective, NA 0.7). To perform embryo dissociation of *P. regularis*, fertilization envelopes were removed mechanically at 1-cell stage, embryos were raised until the 8-cell stage and passed through a 60-μm nylon mesh. Individual blastomeres were raised for 48h at 20°C and imaged.

### Statistical analysis

Statistical analyses of data were performed using Python scripts as indicated in the figure captions. The Rayleigh test was performed to assess bias in the positions of labelled clones following lineage tracing experiments in Figs. 2, 3 and 4. No statistical method was used to predetermine sample size, the experiments were not randomized and the investigators were not blinded to allocation during experiments and outcome assessment.

## Supplementary Information

**Additional file 1: Fig S1.** Cell size asymmetries in asteroid sea star embryos. Representative DIC images of 16-cell stage embryos of *P. miniata* (A) and *P. regularis* (B). Arrowheads point at small cells and arrows point at large cells. Scale bars: 50 μm.

**Additional file 2: Fig S2.** 3D reconstructions of early echinoderm embryos. Representative images of sea urchin (*Lytechinus pictus*) and sea stars (*Patiria miniata*) embryos at the 4, 8 and 16 cells stages. Embryos were injected with mRNA coding for a membrane bound YFP (mYFP) and fluorescently tagged histone (H2B-RFP) and subsequently imaged live on a confocal microscope. The datasets were segmented using the Fiji plugin Limeseg and individual blastomeres rendered as 3D meshes. Scale bars: 50 μm.

**Additional file 3: Movie S1.** Early *Lytechinus Pictus* embryo. 3D rendering video of a live imaged sea urchin embryo (*Lytechinus pictus*) Embryos were injected with mRNA coding for a membrane bound fluorescent protein (mYFP, yellow) and fluorescently tagged histone (H2B-RFP, cian) and subsequently imaged live on a confocal microscope. A lateral view is shown, animal side on top.

**Additional file 4: Movie S2.** Early *Patiria miniata* embryo. 3D rendering video of a live imaged sea star embryo (*Patiria miniata*) Oocytes were injected with mRNA coding for a membrane bound fluorescent protein (mYFP, yellow) and fluorescently tagged histone (H2B-RFP, cian), incubated ON at 16C and subsequently activated, fertilized and imaged live on a confocal microscope. A lateral view is shown, animal side on top.

**Additional file 5: Movie S3.** Early *Patiriella regularis* embryo. 3D rendering video of a live imaged sea star embryo (*Patiriella regularis*) Oocytes were injected with mRNA coding for a membrane bound fluorescent protein (mGFP, yellow) and fluorescently tagged histone (H2A-mCherry, cian), incubated ON at 16C and subsequently activated, fertilized and imaged live on a confocal microscope. A lateral view is shown, animal side on top.

**Additional file 6: Fig S3.** First and third cleavage predict the anteroposterior axis in *P. regularis* sea star embryo. (A-D) *P. regularis* embryos injected with a lineage tracer at the 2-cells stage. One blastomere was injected with mRNA coding for Histone-RFP at the 2-cells stage. Embryos were then raised at 20C, fixed, stained with Draq5 (nuclei) and imaged in toto on a confocal microscope at gastrula and bipinnaria stages. (A) Representative image of an injected embryo at the gastrula stage. (B) Alignment of the first cleavage with the animal-vegetal axis. Images of gastrula stage embryos were rendered in 3D and the angle formed between the clone formed by the injected blastomere and the animal-vegetal axis was measured. n= 8 embryos. (C) Representative image of an injected embryo at the bipinnaria stage. (D) Alignment of the first cleavage with the DV axis. Images of bipinnaria stage embryos were rendered in 3D and the angle formed between the clone formed by the injected blastomere and the sagittal plane was measured. n= 15 embryos. (E) Representative images of *P. regularis* embryos injected with a lineage tracer at the 8-cells stage. One blastomere was injected with mRNA coding for Histone-RFP at the 8-cells stage, embryos were then raised at 20C until gastrula stage, fixed, stained with Draq5 (nuclei) and imaged in toto on a confocal microscope. Two types of clones were observed, either forming anterior ectoderm or posterior ectoderm and mesendoderm tissues. (F) Representative images of *P. regularis* embryos formed by individual blastomeres separated at the 8-cells stage. Fertilization envelopes were removed mechanically at 1-cell stage, embryos were raised until the 8-cells stage and dissociated by passing them through a 60 μm nylon mesh. Individual blastomeres were raised for 48h at 20C. Two types of embryos were observed, blastulae and gastrulae. Scale bars: 50 μm.

**Additional file 7: Fig S4.** Photoconversion of Kaede expressing sea star embryos. Representative confocal images of a photoconversion experiment. P. miniata oocytes were injected with mRNA coding for the photoconvertible protein Kaede (Kaede) and incubated ON at 16C. Oocytes were subsequently activated, fertilized and incubated until 16 cells stage, when one of the 16-cells was photoconverted (pcKaede) on a confocal microscope (405 nm laser). (A) 3D rendering of a 16-cells stage embryo before photoconversion. (B) Close up images of the photoconverted cell after 0, 30 and 60 seconds of exposure to a 405 nm laser. (C) 3D rendering of the same embryo after photoconversion. Scale bars: 50 μm.

**Additional file 8: Fig S5.** *P. miniata* lineage tracing at 16-cells stage: photoconversion of one random cell. Schematic representations of sea star larvae showing the clone derived by one cell at 16 cells stage. Oocytes were injected with mRNA coding for the photoconvertible protein Kaede (Kaede) and incubated ON at 16C. Oocytes were subsequently activated, fertilized and incubated until 16 cells stage, when one of the 16 cells was photoconverted (Kaede) on a confocal microscope (405 nm laser). Embryos were raised at 16C for 72 hpf and then imaged live in toto on a confocal microscope. Images were 3D rendered and schematic representations of the clones were drawn for easier comparison. Each clone (rows) is represented by dorsal, ventral and lateral views and distinguishing between ectodermal and mesendodermal tissues. 

**Additional file 9: Fig S6.** *P. miniata* lineage tracing at 16-cells stage: photoconversion of one small cell. Schematic representations of sea star larvae showing the clone derived by one small cell at 16 cells stage. Oocytes were injected with mRNA coding for the photoconvertible protein Kaede (Kaede) and incubated ON at 16C. Oocytes were subsequently activated, fertilized and incubated until 16 cells stage, when the smallest of the 16 cells was photoconverted (Kaede) on a confocal microscope (405 nm laser). Embryos were raised at 16C for 72 hpf and then imaged live in toto on a confocal microscope. Images were 3D rendered and schematic representations of the clones were drawn for easier comparison. Each clone (rows) is represented by dorsal, ventral and lateral views and distinguishing between ectodermal and mesendodermal tissues.

**Additional file 10: Movie S4.** Photoconverted *P. miniata* larva. Animation showing a 3D reconstruction of a representative embryo photoconverted at the 16-cells stage. Oocytes were injected with mRNA coding for the photoconvertible protein Kaede (green) and incubated ON at 16C. Oocytes were subsequently activated, fertilized and incubated until 16-cell stage, when one of the 16-cell was photoconverted (red) on a confocal microscope (405 nm laser). Embryos were raised at 16C for 72 hpf and then imaged live in toto on a confocal microscope. Images were 3D rendered with Imaris (Bitplane) and animation rotating the dataset was recorded. Kaede is shown in green and photoconverted Kaede in red.

**Additional file 11: Fig S7.** P. miniata lineage tracing of animal cells. Schematic representations of sea star larvae in which one animal cell was injected at the 8-cell stage. Oocytes were injected with H2B-CFP to mark nuclei, fertilized and raised until the 8-cell stage, when one animal blastomere was injected with DiI. Embryos were imaged in toto on a confocal microscope at three different developmental stages (26, 50 and 72 hpf). Images were 3D rendered and schematic representations of the clones were drawn for easier comparison. Each clone (rows) is represented at the three developmental stages and by dorsal, ventral and lateral views of the ectodermal tissues. Given that the dorsoventral axis cannot be identified at the 26 hpf stage, a frontal view of the labelled clone is provided and a section to show the animal/vegetal domain covered by the clone.

**Additional file 12: Fig S8.** *P. miniata* blastomere dissociations. Representative DIC images of larvae generated by blastomeres of dissociated embryos. Embryos were either not manipulated (CTRL) or dissociated at the 2-, 4-, or 8-cells stage. Individual blastomeres generated by dissociation at the 2-, 4-, and 8-cells stage, and animal and vegetal quartets generated by halving embryos at the 8-cells stage were raised until 120 hpf. A subset of embryos was live imaged at 48, 72 and 120 hpf. Vegetal or animal identity was established according to the position of the polar bodies. Scale bars: 100 μm.

## Data Availability

All microscopy data is freely available for download at “Barone, Vanessa; Byrne, Maria; Lyons, Deirdre C. (2022). Data from: Lineage tracing shows that cell size asymmetries predict the dorsoventral axis in the sea star embryo. UC San Diego Library Digital Collections. 10.6075/J02F7NNK”. Materials that are not commercially available will be provided upon request.
