# Supplementary figures and images for: Lineage tracing shows that cell size asymmetries predict the dorsoventral axis in the sea star embryo
Source: BMC Biol. 2022 Aug 15;20:179. doi: 10.1186/s12915-022-01359-3 (PMC9380389; doi:10.1186/s12915-022-01359-3)

*P. miniata*

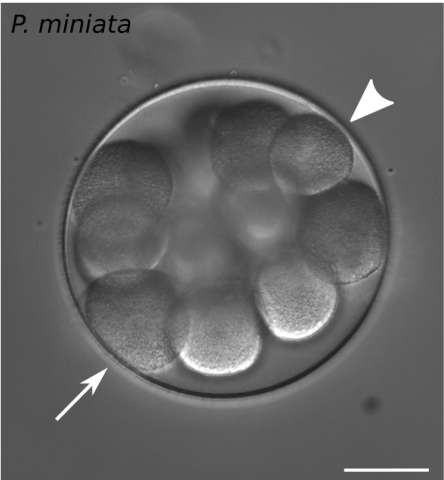

*P. regularis*

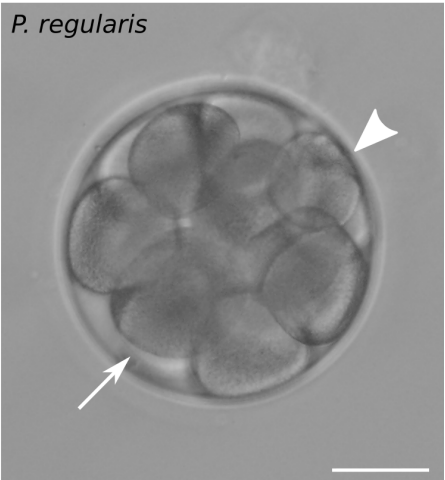

Supplement: Supplementary file 1 — Additional file 1: Fig S1. Cell size asymmetries in asteroid sea star embryos. Representative DIC images of 16-cell stage embryos of P. miniata (A) and P. regularis (B). Arrowheads point at small cells and arrows point at large cells. Scale bars: 50 μm. [file 12915_2022_1359_MOESM1_ESM.pdf]

*Lytechinus pictus*

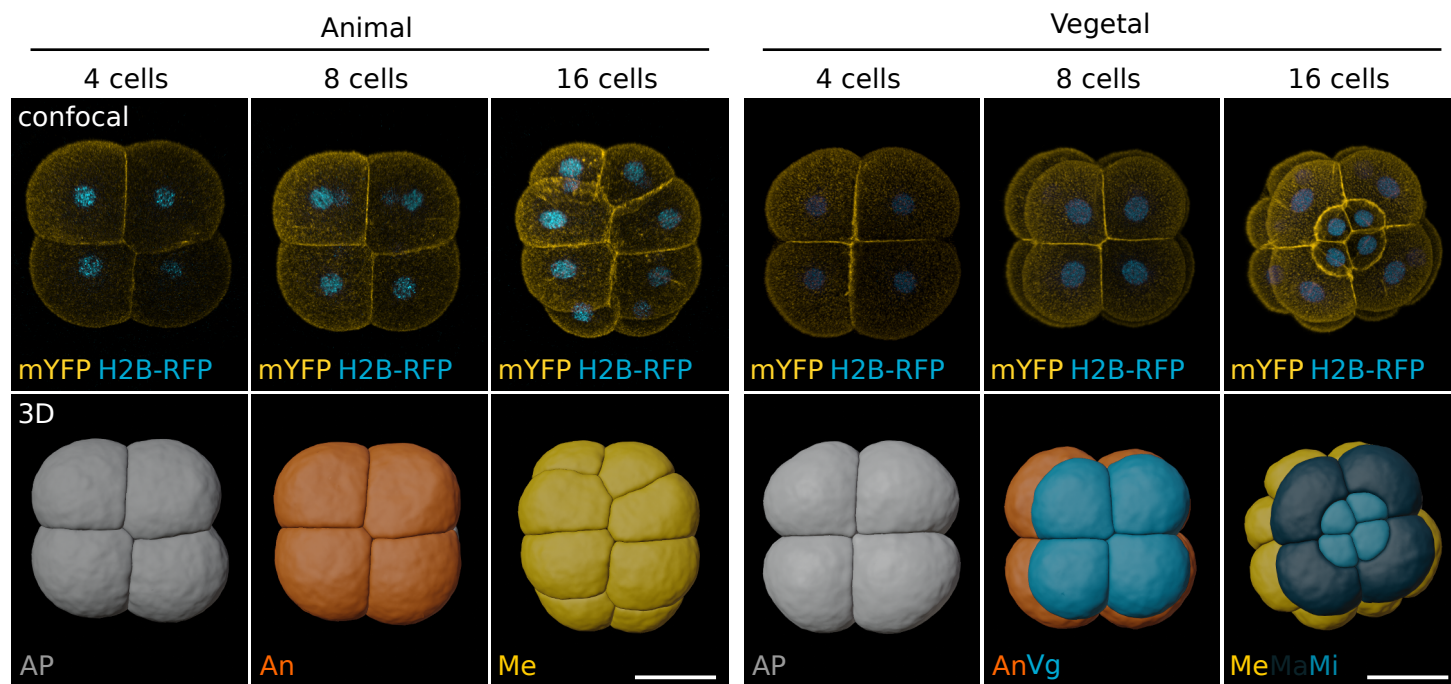

*Patiria miniata*

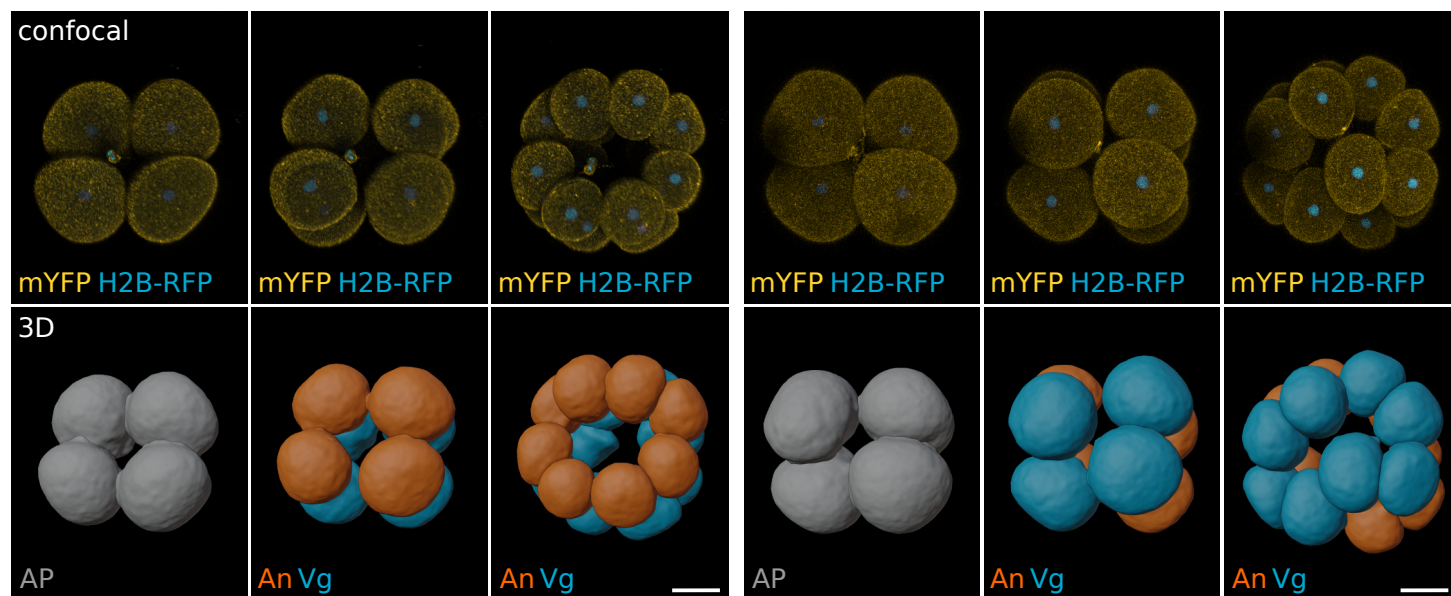

Supplement: Supplementary file 2 — Additional file 2: Fig S2. 3D reconstructions of early echinoderm embryos. Representative images of sea urchin (Lytechinus pictus) and sea stars (Patiria miniata) embryos at the 4, 8 and 16 cells stages. Embryos were injected with mRNA coding for a membrane bound YFP (mYFP) and fluorescently tagged histone (H2B-RFP) and subsequently imaged live on a confocal microscope. The datasets were segmented using the Fiji plugin Limeseg and individual blastomeres rendered as 3D meshes. Scale bars: 50 μm. [file 12915_2022_1359_MOESM2_ESM.pdf]

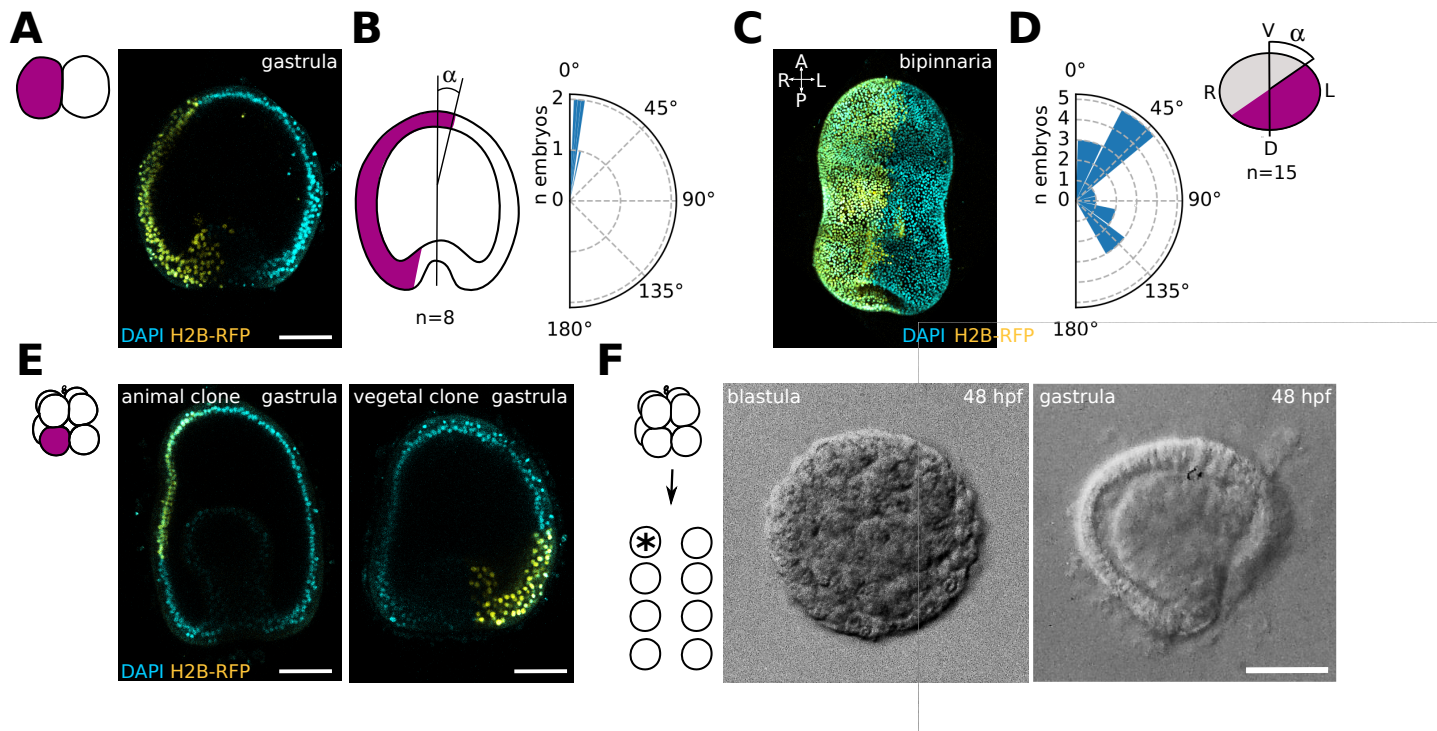

Supplement: Supplementary file 6 — Additional file 6: Fig S3. First and third cleavage predict the anteroposterior axis in P. regularis sea star embryo. (A-D) P. regularis embryos injected with a lineage tracer at the 2-cells stage. One blastomere was injected with mRNA coding for Histone-RFP at the 2-cells stage. Embryos were then raised at 20C, fixed, stained with Draq5 (nuclei) and imaged in toto on a confocal microscope at gastrula and bipinnaria stages. (A) Representative image of an injected embryo at the gastrula stage. (B) Alignment of the first cleavage with the animal-vegetal axis. Images of gastrula stage embryos were rendered in 3D and the angle formed between the clone formed by the injected blastomere and the animal-vegetal axis was measured. n= 8 embryos. (C) Representative image of an injected embryo at the bipinnaria stage. (D) Alignment of the first cleavage with the DV axis. Images of bipinnaria stage embryos were rendered in 3D and the angle formed between the clone formed by the injected blastomere and the sagittal plane was measured. n= 15 embryos. (E) Representative images of P. regularis embryos injected with a lineage tracer at the 8-cells stage. One blastomere was injected with mRNA coding for Histone-RFP at the 8-cells stage, embryos were then raised at 20C until gastrula stage, fixed, stained with Draq5 (nuclei) and imaged in toto on a confocal microscope. Two types of clones were observed, either forming anterior ectoderm or posterior ectoderm and mesendoderm tissues. (F) Representative images of P. regularis embryos formed by individual blastomeres separated at the 8-cells stage. Fertilization envelopes were removed mechanically at 1-cell stage, embryos were raised until the 8-cells stage and dissociated by passing them through a 60 μm nylon mesh. Individual blastomeres were raised for 48h at 20C. Two types of embryos were observed, blastulae and gastrulae. Scale bars: 50 μm. [file 12915_2022_1359_MOESM6_ESM.pdf]

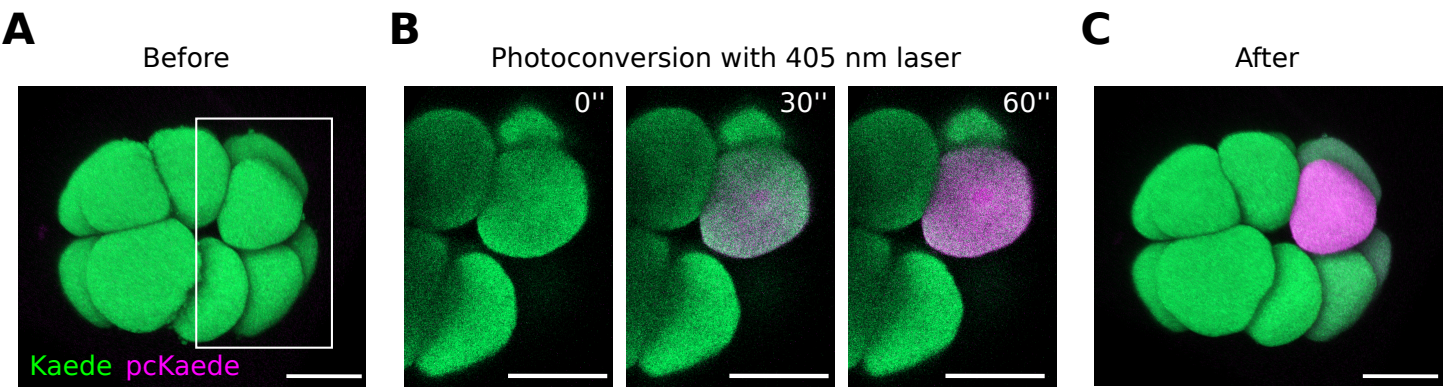

Supplement: Supplementary file 7 — Additional file 7: Fig S4. Photoconversion of Kaede expressing sea star embryos. Representative confocal images of a photoconversion experiment. P. miniata oocytes were injected with mRNA coding for the photoconvertible protein Kaede (Kaede) and incubated ON at 16C. Oocytes were subsequently activated, fertilized and incubated until 16 cells stage, when one of the 16-cells was photoconverted (pcKaede) on a confocal microscope (405 nm laser). (A) 3D rendering of a 16-cells stage embryo before photoconversion. (B) Close up images of the photoconverted cell after 0, 30 and 60 seconds of exposure to a 405 nm laser. (C) 3D rendering of the same embryo after photoconversion. Scale bars: 50 μm. [file 12915_2022_1359_MOESM7_ESM.pdf]

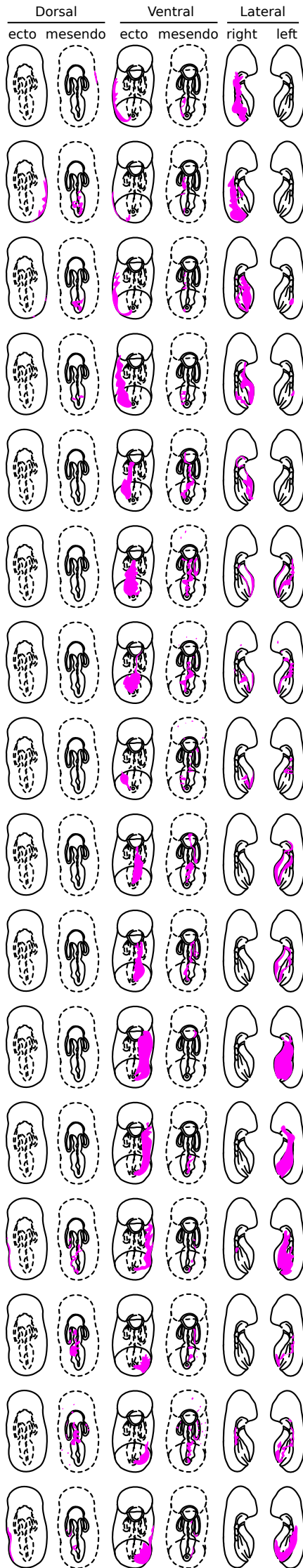

Supplement: Supplementary file 8 — Additional file 8: Fig S5. P. miniata lineage tracing at 16-cells stage: photoconversion of one random cell. Schematic representations of sea star larvae showing the clone derived by one cell at 16 cells stage. Oocytes were injected with mRNA coding for the photoconvertible protein Kaede (Kaede) and incubated ON at 16C. Oocytes were subsequently activated, fertilized and incubated until 16 cells stage, when one of the 16 cells was photoconverted (Kaede) on a confocal microscope (405 nm laser). Embryos were raised at 16C for 72 hpf and then imaged live in toto on a confocal microscope. Images were 3D rendered and schematic representations of the clones were drawn for easier comparison. Each clone (rows) is represented by dorsal, ventral and lateral views and distinguishing between ectodermal and mesendodermal tissues. [file 12915_2022_1359_MOESM8_ESM.pdf]

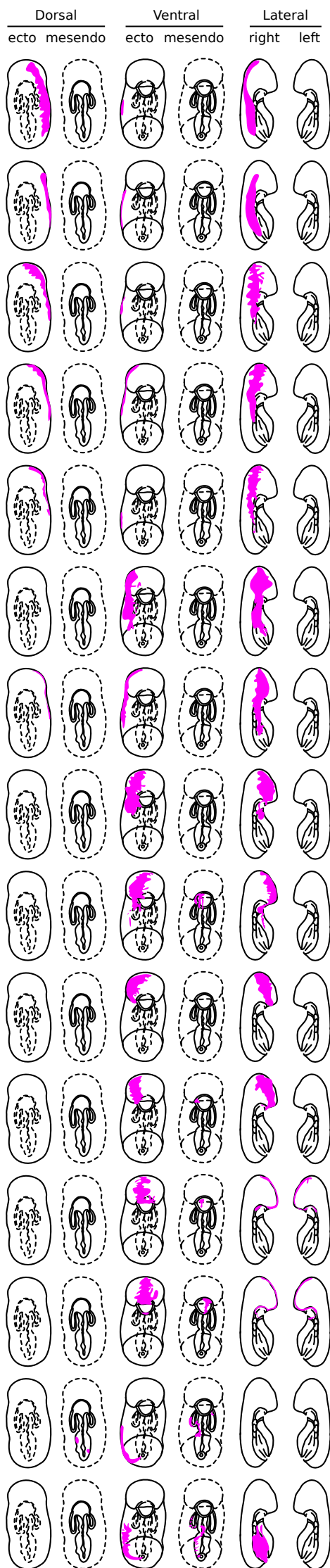

Supplement: Supplementary file 9 — Additional file 9: Fig S6. P. miniata lineage tracing at 16-cells stage: photoconversion of one small cell. Schematic representations of sea star larvae showing the clone derived by one small cell at 16 cells stage. Oocytes were injected with mRNA coding for the photoconvertible protein Kaede (Kaede) and incubated ON at 16C. Oocytes were subsequently activated, fertilized and incubated until 16 cells stage, when the smallest of the 16 cells was photoconverted (Kaede) on a confocal microscope (405 nm laser). Embryos were raised at 16C for 72 hpf and then imaged live in toto on a confocal microscope. Images were 3D rendered and schematic representations of the clones were drawn for easier comparison. Each clone (rows) is represented by dorsal, ventral and lateral views and distinguishing between ectodermal and mesendodermal tissues. [file 12915_2022_1359_MOESM9_ESM.pdf]

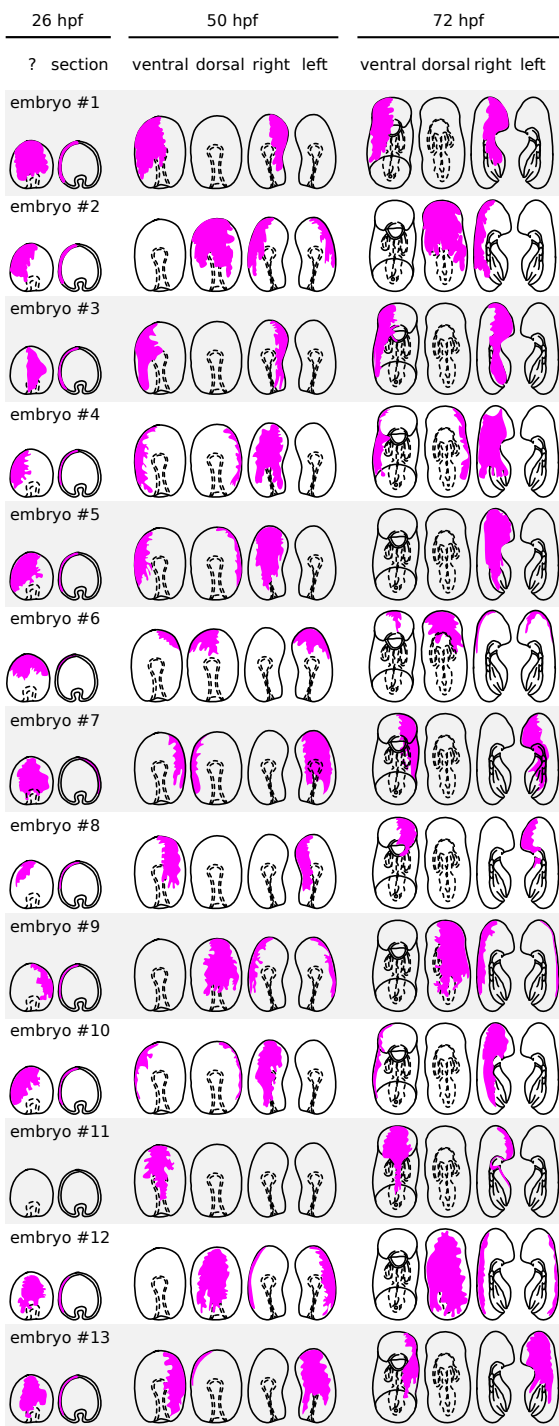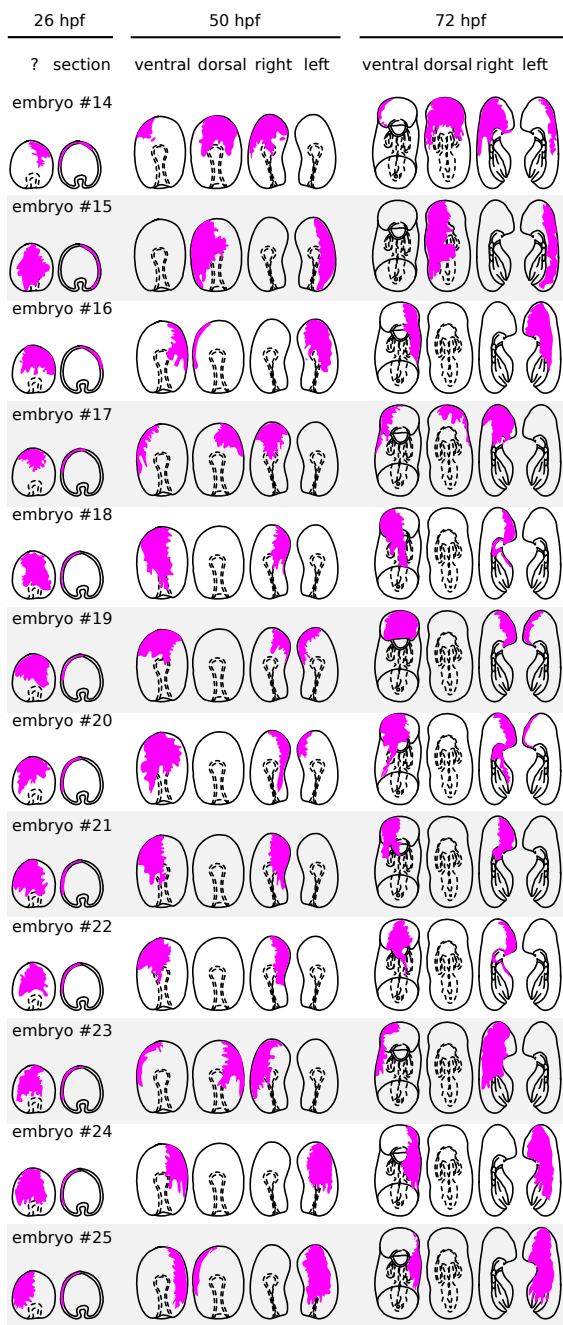

Supplement: Supplementary file 11 — Additional file 11: Fig S7. P. miniata lineage tracing of animal cells. Schematic representations of sea star larvae in which one animal cell was injected at the 8-cell stage. Oocytes were injected with H2B-CFP to mark nuclei, fertilized and raised until the 8-cell stage, when one animal blastomere was injected with DiI. Embryos were imaged in toto on a confocal microscope at three different developmental stages (26, 50 and 72 hpf). Images were 3D rendered and schematic representations of the clones were drawn for easier comparison. Each clone (rows) is represented at the three developmental stages and by dorsal, ventral and lateral views of the ectodermal tissues. Given that the dorsoventral axis cannot be identified at the 26 hpf stage, a frontal view of the labelled clone is provided and a section to show the animal/vegetal domain covered by the clone. [file 12915_2022_1359_MOESM11_ESM.pdf]

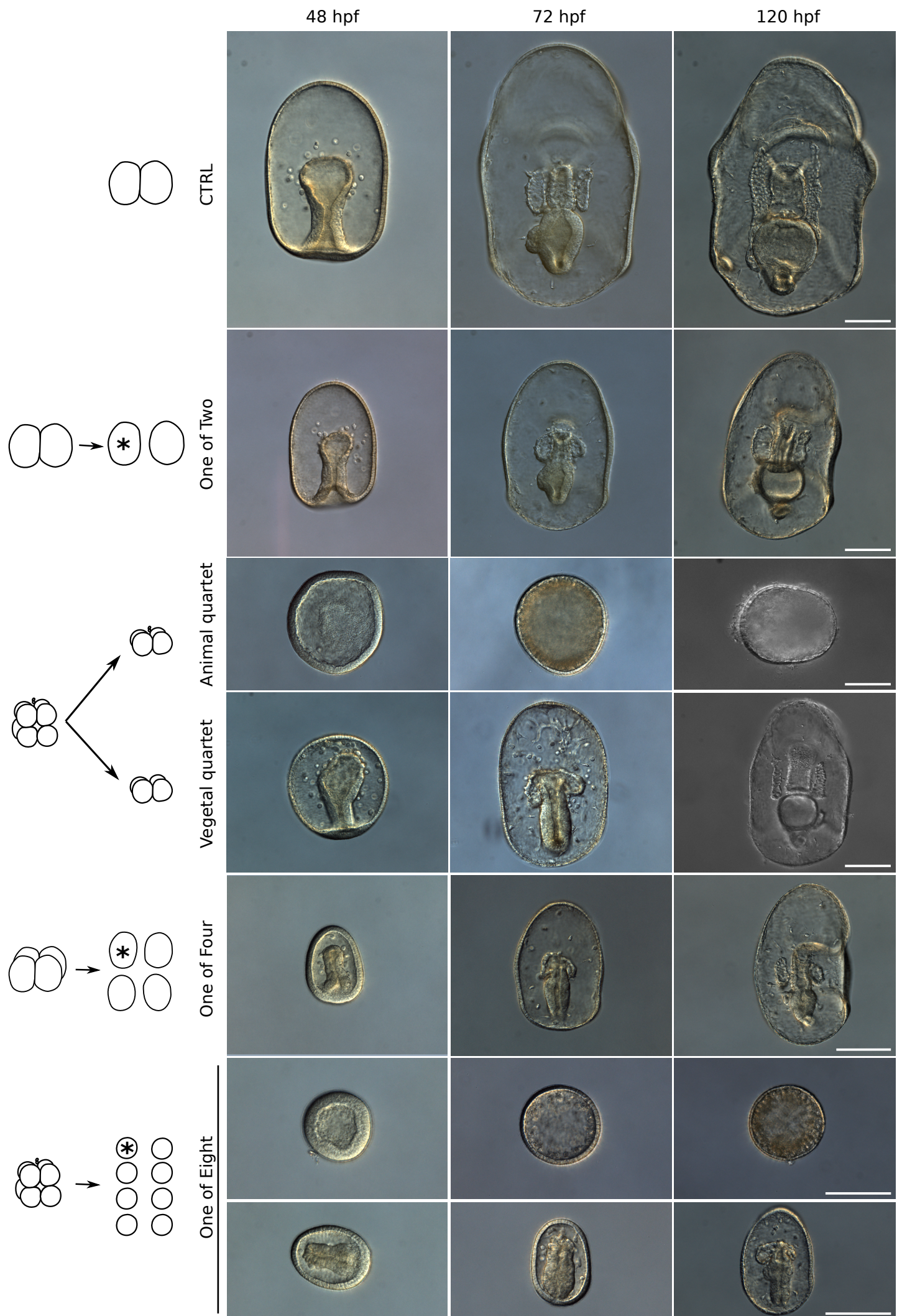

Supplement: Supplementary file 12 — Additional file 12: Fig S8. P. miniata blastomere dissociations. Representative DIC images of larvae generated by blastomeres of dissociated embryos. Embryos were either not manipulated (CTRL) or dissociated at the 2-, 4-, or 8-cells stage. Individual blastomeres generated by dissociation at the 2-, 4-, and 8-cells stage, and animal and vegetal quartets generated by halving embryos at the 8-cells stage were raised until 120 hpf. A subset of embryos was live imaged at 48, 72 and 120 hpf. Vegetal or animal identity was established according to the position of the polar bodies. Scale bars: 100 μm. [file 12915_2022_1359_MOESM12_ESM.pdf]
